# Developmental Outcomes at 6 Months in Neonates with First-minute Apgar Score Below 7 and Gestational Age More Than 35 Weeks: A Single-center Study: Developmental Outcomes in Neonates with Low First-minute Apgar

**DOI:** 10.31661/gmj.v14i.3838

**Published:** 2025-11-05

**Authors:** Shaghayegh Moradi Alamdarloo, Fereshteh Nassari, Atefe Hashemi, Elham askary, Roya Mohammadi, Khadijeh Bazrafshan, Leila Kiani, Hamide Barzegar

**Affiliations:** ^1^ Maternal-Fetal Medicine Research Center, Department of Obstetrics and Gynecology, School of Medicine, Shiraz University of Medical Sciences, Shiraz, Iran; ^2^ Department of Obstetrics and Gynecology, Maternal-Fetal Medicine Research Center, School of Medicine, Shiraz University of Medical Sciences, Shiraz, Iran; ^3^ Children’s Medical Center, Tehran University of Medical Sciences, Tehran, Iran; ^4^ Neonatal Research Center, Shiraz University of Medical Sciences, Shiraz, Iran

**Keywords:** Apgar Score, Growth and Development, Newborn; Infant

## Abstract

**Background:**

Background: The objective of this study was to investigate the relationship between the one-minute Apgar score and developmental outcomes at six months of age.

**Materials and Methods:**

Materials and Methods: This cohort study involved neonates with gestational age of more than 35 weeks and a first-minute Apgar score of less than 7, without major anomalies requiring admission during September 2018-2021. Clinical data and hospital course details were recorded. At 6 months old, participants were contacted, and parental questionnaires regarding age and stages questionnaire (ASQ) were completed. Data were analyzed by SPSS 21, using mean, standard deviation, frequency, and percentage. Dunn test and the Kruskal-Wallis test were also employed.

**Results:**

Results: Out of 26,928 neonates born, 865 had an Apgar score of less than 7 and survived the first day, with 208 being more than 35 weeks gestational age and needing admission. Ultimately, 196 neonates were enrolled. The mean gestational age was 38.2± 1.75 and the mean 1st-minute Apgar score was 3.36 ± 1.84. A significant relationship was observed between the 5th-minute Apgar score and cord blood acidosis (P0.001) and the 10th-minute Apgar score with the need for oxygen (P=0.02(. Most infants had normal ASQ evaluations, while one (0.5%) was in high-risk zone for each domain of fine motor, personal-social, and communication skills. Additionally, one (0.05%) was in the abnormal range for gross motor function, and another one (0.05%) was in the abnormal range for fine motor function.

**Conclusion:**

Conclusion: Our study did not identify a relationship between a low first-minute Apgar score and adverse developmental outcomes at 6 months of age.

## Introduction

The Apgar score, developed by Dr. Virginia Apgar in 1952, remains one of the most widely used tools for the rapid assessment of newborns immediately after birth [1]. It consists of five parameters—skin color, heart rate, reflex irritability, muscle tone, and respiratory effort—each scored from 0 to 2, leading to a total score ranging from 0 to 10. Although originally created to guide the need for immediate postnatal intervention, particularly at the first minute of life, it has since become a key indicator of neonatal well-being and is used worldwide [2]. While the Apgar score was not designed to predict long-term health outcomes or serve as a definitive diagnostic tool, growing evidence suggests a relationship between Apgar scores and later neurodevelopmental status. For instance, Odd et al. (2008) demonstrated a significant association between lower Apgar scores at five minutes and poorer cognitive performance in childhood [3]. Similarly, studies have reported that low Apgar scores correlate with an increased risk of language impairments [4]and educational difficulties during school years [5]. These findings suggest that the Apgar score may have broader implications beyond the immediate postnatal period.

Despite these insights, most prior research has focused on the Apgar score at the fifth or tenth minute, potentially underestimating the clinical relevance of the first-minute score. Many studies either neglect the one-minute Apgar altogether or interpret it merely as a transient finding that improves after initial resuscitation [6].

However, the first-minute Apgar score might reflect more than just temporary distress—it could indicate subtle perinatal insults or compromised neonatal adaptation that may influence early development [6][7].

Yet, very few studies have examined how the first-minute Apgar score alone might relate to short-term developmental milestones, especially in infancy. In this study, we aim to address this gap by investigating the relationship between first-minute Apgar scores and developmental outcomes up to six months of age. By focusing specifically on the earliest Apgar assessment, we aim to investigate whether even mild deviations from a perfect score at one minute have significant implications during early infancy. This work not only builds on existing evidence but also offers a novel perspective by evaluating outcomes during a critical window of neurodevelopment that has been largely overlooked in prior literature.

## Materials and Methods

This cohort study was conducted among neonates born at 35 weeks gestational age or more, with a 1st-minute Apgar score of less than 7, at Hazrat Zeinab Hospital in Shiraz, between September 2018 and September 2021. Neonates with major anomalies at birth were excluded from the study, resulting in a final enrollment of 196 neonates. Demographic and hospital information, details such as gestational age, birth weight, and Apgar scores at 1, 5, and 10 minutes after birth were included, respiratory and cardiac status, need for echocardiography or brain sonography, and any complications during admission, were obtained from hospital records. Parents were contacted through telephone and asked to complete the Ages and Stages Questionnaire (ASQ) form at 6 months old. The ASQ is a validated questionnaire used for screening the development of infants and children aged 2 months to 60 months [8][9]. It assesses various domains of development encompassing areas such as personal-social development, gross and fine motor abilities, problem-solving capacity, and communication skills [8]. Its reliability and validity in Persian was confirmed [10].

In the descriptive section of this study, mean, standard deviation, frequencies, and percentages were reported. The normality of Apgar scores at 1st, 5th, and 10th minutes was assessed using the Kolmogorov-Smirnov test. For comparing means between two groups the Mann-Whitney U test was employed. Evaluation and comparison of means among three or more groups were conducted using a non-parametric counterpart, the Kruskal-Wallis test. The Dunn test was employed for pairwise comparisons between the groups. Data analysis was performed using Statistical Package for the Social Sciences (SPSS ) version 21 (IBM Corp., Armonk, NY, USA), with a baseline significance level set at 0.05. The correlation between quantitative factors was assessed using either the Pearson correlation test or its non-parametric equivalent, Spearman's rho correlation test.

### Ethical Statements

The research protocol adhered to the ethical principles outlined in the Declaration of Helsinki (1975). Approval for the publication was granted by the Ethics Committee of Shiraz University of Medical Sciences. All methods were conducted in accordance with applicable guidelines and regulations. Informed consent was obtained from the parents. The study protocol was reviewed and approved by the Medical Ethics Committee of Shiraz University of Medical Sciences (Ethics Code: IR.SUMS.MED.REC.1400.164).

## Results

**Table:** Table1. Demographic and Clinical Data of Mothers and Neonates

		**Mean ± SD**
**Maternal age**		30.11 ± 6.29
**Number of pregnancies**		2.51 ± 1.51
**Number of Live births**		1.17 ± 1.33
**Number of abortions**		0.34 ±0.59
**Gestational age**		38.2± 1.75
**Dilatation (cm)**		2.54 ± 2.71
**Duration of induction (min)**		186.65 ± 291.72
	**PH**	7.24 ± 0.16
**Cord blood gas**	**PCO2**	47.04 ± 17.28
	**HCO3**	20.64 ± 6.41
	**BE**	-7.15 ± 6.27
	**1^st^ min. **	3.36 ± 1.84
**Apgar Score**	**5^th^ min. **	6.36 ± 1.62
	**10^th^ min. **	7.88 ± 1.63
**Birth weight**		3090.18 ± 649.64
**Oxygen support duration**	**Oxygen**	16.05 ± 32.72
**CPAP**	8.61 ± 32.61
**Intubation**	23.05 ± 127.31

PH: potential of hydrogen, PCO2: partial pressure of carbon dioxide, BE: base excess, CPAP: continuous positive airway pressure

**Table:** Table2. The Relationship between Prenatal and Natal Factors with Apgar Scores

	**Apgar score**		
	**1^st^ min. **	**5^th^ min. **	**10^th^ min. **
**Gestational age**	r: -0.006 p: 0.942	r: 0.186 p: 0.01	r: 0.198 p: 0.005
**Dilatation**	r: -0.133 p: 0.063	r: -0.173 p: 0.015	r: -0.062 p: 0.38
**PH**	r: 0.165 p: 0.148	r: 0.298 p: <0.001	r: 0.129 p: 0.124
**HCO3**	r: 0.051 p: 0.55	r: 0.195 p: 0.02	r: 0.1 p: 0.23
**BE**	r: 0.08 p: 0.034	r: 0.18 p: 0.032	r: 0.069 p: 0.411
**Duration of hospitalization**	r: -0.278 p: <0.001	r: -0.382 p: <0.001	r: -0.278 p: <0.001
**Intubation**	r: -0.169 p: 0.347	r: -0.301 p: 0.088	r: -0.384 p: 0.028
**Oxygen delivery after birth**	r: -0.014 p: 0.892	r: -0.128 p: 0.208	r: -0.231 p: 0.021
**Birth weight**	r: 0.093 p: 0.194	r: 0.216 p: 0.002	r: 0.26 p: <0.001

^*^Assessed by Spearman correlation test. P-value <0.05 is considered significant

**Table:** Table3. Neonatal Outcomes and Apgar Score

**Apgar**	**Group comparison**	**Statistical index**	P-value*
**1 ^st^ minute **	Transfer-dead Stable-dead Stable-transfer	3.87 4.2 4.75	0.148 0.079 0.088
**5 ^th^ minute **	Transfer-dead Stable-dead Stable-transfer	12.01 1.21 4.75	0.002 0.81 0.088
**10 ^th^ minute **	Transfer-dead Stable-dead Stable-transfer	8.44 10.15 1.31	0.011 0.004 0.755

^*^Assesed by Dunn Post-hoc Test. P-value less than 0.05 considered significant

**Table:** Table4. Infant’s Evaluation in Different Domains of ASQ

	**Normal**	**In the risk zone**	**Abnormal**
**Gross motor**	195 (99.5%)	0	1(0.5%)
**Fine motor**	194(99%)	1(0.5%)	1(0.5%)
**Problem-solving**	196(100%)	0	0
**Personal Social**	195(99.5%)	1(0.5%)	0
**Communication**	195(99.5%)	1(0.5%)	0

During the study period, 26,928 neonates were born. Of these, 865 neonates had a first-minute Apgar score of less than 7 and survived the first day. Among them, 208 neonates were more than 35 weeks gestational age and needed admission. Ultimately, 196 neonates met the criteria and were enrolled in the study, among whom 21(10.8%) neonates died during the study period. The mode of delivery was vaginal in 57 (29%) cases and via cesarean section in 139 (71%). Perinatal sonography revealed normal findings in 183 (94%) cases, while the remaining cases showed various abnormalities including hydrops fetalis, polyhydramnios, intrauterine growth retardation, cardiac anomalies, and cleft palate. Cesarean sections were performed due to elective reasons in 11 (6%) cases, previous section and labor pain in 17 (9%) cases, preeclampsia or eclampsia in 22 (11%) cases, meconium staining in 16 (8%) cases, cord prolapse in 5 (2.5%) cases, full arrest in 5 (2.5%) cases, abnormal presentation in 25 (13%) cases, severe vaginal bleeding in 7 (3.5%) cases, and fetal distress in 26 (13%) cases. Non-stress tests (NST) showed reactive results in 120 (61%) cases, non-reactive in 45 (23%) cases, and were unknown in 31 (16%) cases.

Demographic data regarding mothers and neonates are summarized in Table-1.

Table-2 illustrates the relationship between prenatal and natal factors with Apgar Scores.

Echocardiography was required for 47 (23.9%) neonates, out of which 30 (63.8%) were within normal limits. The abnormalities observed included ventricular septal defect, pulmonary hypertension, cardiomegaly, and tricuspid regurgitation. The P-values for the relationship between the need for echocardiography and Apgar scores at the 1st, 5th, and 10th minutes were 0.344, 0.001, and 0.019, respectively.

Additionally, 64 (32.6%) neonates required brain sonography. The P-values for the relationship between the need for sonography and Apgar scores at 1st, 5th, and 10th minutes were 0.009, <0.001, and <0.001, respectively.

The relationship between Apgar scores and the condition of fetal membranes (intact, meconium, ruptured, bleeding) was found to be insignificant (P>0.05). Similarly, the relationship between Apgar scores and NST, as well as different fetal positions, was also insignificant (P>0.05).

A multiple linear regression analysis was conducted to examine the association between maternal age and neonatal Apgar scores at 1, 5, and 10 minutes, controlling for gravidity, induction time, type of delivery, and CPAP requirement. The model predicting Apgar score at 1 minute was statistically significant (F(5, 111)=5.03, P<0.001). Maternal age was significantly associated with Apgar score at 1 minute (B=0.077, SE=0.034, β=0.233, P=0.027), suggesting that each additional year of maternal age was associated with a 0.077 point increase in Apgar score. CPAP requirement had a significant negative association with Apgar score (B=-1.040, P=0.001). Gravidity, induction time, and type of delivery were not significant predictors (P=0.335, 0.272, and 0.161, respectively). For the Apgar score at 5 minutes, the model was also significant (F(5, 111)=3.98, P=0.002), explaining 15.2% of the variance (R²=0.152). Maternal age was not a significant predictor (B=0.051, P=0.087). CPAP requirement remained a significant negative predictor (B=-0.739, P=0.002). Other covariates were not statistically significant (gravidity: P=0.496, induction time: P=0.681, type of delivery: P=0.281). The regression model for Apgar score at 10 minutes was not statistically significant (F(5, 111)=1.77, P=0.126*, R²=0.074). Neither maternal age (P=0.182) nor the other variables significantly predicted the Apgar score at this time point.

Mean Apgar scores at the 5th and 10th minutes were significantly higher in surviving neonates compared to those who died (P<0.001).At birth, respiratory status was normal in 55 (28%) neonates, while 79 (41%) required oxygen support, 31 (15.8%) required continuous positive airway pressure, and 30 (15.3%) required intubation.

Admissions were primarily due to low Apgar scores in 106 (54%) cases, prematurity and low Apgar scores in 80 (41%) cases, and other complications in 3 (1.5%) cases. 98 (50%) neonates had no complications or complaints during hospital admission, while others presented with various issues such as neonatal jaundice, cardiorespiratory arrest, asphyxia, sepsis, cardiac problems, respiratory problems, and other complications. Ultimately, 170 (87%) neonates were discharged in good condition, 5 (2.5%) were transferred to other hospitals, and 21 (10.5%) died during the study period. Table-3 shows the comparison between these groups.

Table-4 presents the ASQ parameters recorded during the follow-up of these neonates.

## Discussion

The study included 196 neonates with a gestational age of 35 weeks or more who had an Apgar score of less than 7 at the first minute and required hospitalization, most of whom were delivered via cesarean section. A significant correlation was observed between gestational age and Apgar scores at the 5th and 10th minutes. Additionally, pH and HCO3 levels showed a significant association with the 5th-minute Apgar score. Apgar scores also correlated significantly with the duration of hospitalization, with the need for intubation being related to the Apgar score at the 10th minute. Furthermore, the Apgar score at the 10th minute showed an inverse relationship with the need for receiving oxygen. The necessity for brain sonography was significantly associated with Apgar scores, while survival was linked to Apgar scores at both the 5th and 10th minutes. However, the developmental outcomes of neonates at 6 months corrected gestational age were found to not be related to the Apgar score.

In our study, we found no association between adverse developmental outcomes at six months old and the 1st minute Apgar score. In contrast, Razaz et al. assessed infants born at term between 1993 and 2009 and examined their development at 5 years of age. Their findings showed that lower 1-minute and 5-minute Apgar scores across the full spectrum were associated with increased risks of adverse developmental outcomes and special needs at five years of age [11]. The same result was also reported by Leinonen [12]. Razaz et al., in a separate investigation into the connection between the five-minute Apgar score and child development at age 5, discovered an inverse relationship between the score and the probability of developmental vulnerability by that age. Their findings suggest that the five-minute Apgar score could be utilized as a population-level measure to identify developmental risk [13]. Tweed et al. conducted an assessment of short and long-term outcomes as well as the educational status of children in relation to Apgar scores. Their findings unveiled that the 5-minute Apgar score was significantly associated with both developmental outcomes and educational attainment [14]. In a study conducted in Australia, it was found that the 5-minute Apgar score exhibited an inverse correlation with developmental outcomes [15]. Stuart et al. demonstrated that the Apgar score at 5 minutes can have an impact on cognitive function, as evidenced by their evaluation at 16 years old [16]. Most studies have focused on the 5-minute Apgar score and its association with developmental outcomes, with less attention given to the 1-minute Apgar score. This suggests a need for further research in this area. Additionally, there is a need for the assessment of these children over longer periods to obtain more reliable results.

The criteria for asphyxia include metabolic acidosis (pH<7) in cord blood, a persistent Apgar score of 0-3 for longer than five minutes, neurological sequelae, and involvement of multiple organs [17]. As evidenced, the Apgar score holds significance when persistently low beyond 5 minutes. In our study, we also observed an association between acidosis (pH, HCO3, and BE) and a low Apgar score at 5 minutes.

Gestational age presents a limitation in assessing the Apgar score [6], as lower gestational ages tend to correlate with lower Apgar scores and increased levels of acidosis [18]. In our study, we found similar outcomes, with the Apgar scores at both 5 and 10 minutes being correlated with gestational age.

A key strength of this study is its emphasis on the one-minute Apgar score, a topic that has been underexplored in prior studies. Nonetheless, several limitations need to be taken into account. Firstly, the study was carried out at a single center, which may restrict the generalizability of the results. Future multicenter studies with larger sample sizes could yield more comprehensive and reliable findings. Additionally, it's important to note that some neonates with complications, such as those experiencing asphyxia, did not survive the study period. As a result, their developmental outcomes could not be assessed, potentially biasing the results toward lower Apgar scores. The last but not least limitation is that the ASQ was used only at 6 months, while some developmental outcomes may emerge later. Future studies utilizing larger populations and employing case-control platforms, with longer follow-ups are recommended to address these limitations and provide further insights into the relationship between first-minute Apgar scores and neonatal development.

## Conclusion

In this study, we found no significant association between the first-minute Apgar score and adverse developmental outcomes at six months of age. However, Apgar scores at the 5th and 10th minutes were significantly associated with certain clinical parameters, including gestational age, pH levels, need for respiratory support, and survival. These findings suggest that while the first-minute Apgar score provides an initial assessment of neonatal condition, it may not be a reliable predictor of early neurodevelopmental outcomes.

Given that developmental delays may manifest beyond six months, longer follow-up studies are needed to determine whether a low first-minute Apgar score has a delayed impact on cognitive and motor development. Future research should also explore the predictive value of later Apgar scores and integrate more comprehensive neurodevelopmental assessments over time.

## Conflict of Interest

The authors declare that they have no conflict of interest.
